# Tobacco Control and Smoking Cessation–Related Content in Oncology Meetings: A Systematic Scoping Review

**DOI:** 10.1016/j.jtocrr.2025.100874

**Published:** 2025-07-03

**Authors:** Sun Choi, Monisha Chawla, Payton Catherwood, David Chen, Ryan S. Huang, William Boateng, Jennifer Do, Maha Khan, Abdulrahman Alghabban, Naa Kwarley Quartey, Srinivas Raman, Meredith E. Giuliani, William K. Evans, Lawson Eng

**Affiliations:** a Cancer Education Program, Princess Margaret Cancer Centre, Toronto, Ontario, Canada; b Department of Radiation Oncology, Princess Margaret Cancer Centre, Toronto, Ontario, Canada; c Division of Medical Oncology and Hematology, Princess Margaret Cancer Centre, Toronto, Ontario, Canada; d Department of Oncology, McMaster University, Hamilton, Ontario, Canada; e Cancer Care Ontario, Ontario Health, Toronto, Ontario, Canada

**Keywords:** Tobacco control, Smoking cessation, Cancer education, Oncology, Knowledge dissemination

## Abstract

**Introduction:**

Despite the importance of smoking cessation in cancer care, it remains unclear how much tobacco control and smoking-related content (TCSCR) is included in major oncology meetings. Developing an understanding of the amount of content can help to improve education and dissemination of the benefits of smoking cessation in cancer care.

**Methods:**

We performed a scoping review of TCSCR abstracts and educational sessions using online programs and abstract books from 2018 to 2023 for 12 major oncology meetings across different disciplines and disease sites.

**Results:**

A total of 5178 TCSCR content was identified using our search criteria; 421 abstracts and 119 educational sessions met the inclusion criteria. Between 2018 and 2023, the World Cancer Congress (WCC) and the World Conference on Lung Cancer (WCLC) had the highest mean percentage of smoking-related abstracts (WCC: 4.96 ± 3.77%; WCLC: 1.81 ± 0.77%) and educational sessions (WCC: 3.48 ± 1.30%; WCLC: 3.15 ± 1.06%). Among the included abstracts, most (79%) first authors were from high-income countries. Around 39% percent of abstracts focused on tobacco as a cancer risk factor, 34% smoking cessation, and 26% cancer outcomes. Most abstracts were presented as posters (65%), as oral abstracts (27%), or as published abstracts (8%). The distribution of topic focus (*p* = 0.004) and session type (*p* < 0.001) differed between abstracts from high-income and low-middle-income countries.

**Conclusions:**

Despite the importance of smoking cessation in oncology, TCSCR abstracts and educational content are limited at major oncology meetings. Organizers of oncology conferences should be encouraged to explore strategies to include sessions and attract submissions on these topics, particularly from underrepresented regions.

## Introduction

Smoking is a known risk factor for cancer.^1^ Most notably, smoking is strongly associated with lung cancer and is estimated to contribute to 90% of lung cancer-related deaths.^2^^,^^3^ However, many other cancers are associated with tobacco use, including head and neck, bladder, and gastrointestinal cancers.^4^, ^5^, ^6^, ^7^, ^8^

Despite the known association between tobacco use and cancer, many patients continue to use tobacco after their diagnosis.^9^ Continued tobacco use is associated with poorer cancer outcomes, including worse survival, reduced treatment response, increased treatment-related toxicities, risk of second primary cancers, and negative impacts on quality of life.^5^^,^^10^, ^11^, ^12^, ^13^, ^14^ In addition to cancer, there are many other adverse health risks to continued tobacco use, including cardiovascular and respiratory disease, which can impact both cancer and noncancer survivors.^1^^,^^15^, ^16^, ^17^, ^18^ Thus, smoking cessation remains essential to optimize cancer outcomes and to improve overall health for patients living with cancer. This is particularly important given the advances in both early cancer detection and treatment, which have led to improvements in survival and a growing population of cancer survivors.^19^

Despite the central importance of smoking cessation in cancer care and its impact on cancer risk and outcomes, smoking cessation and tobacco control-related content are often not highlighted in oncology meetings. With advances in cancer treatment, including immunotherapy, antibody-drug conjugates and targeted therapies, and new surgical and radiation approaches, there is a need to better understand how tobacco use impacts the outcomes and toxicities of these newer therapies. In addition, emerging health behaviors, including vaping and cannabis use, may potentially impact cancer risk and outcomes, although further research is needed.^20^^,^^21^ Exploring strategies to increase and disseminate research related to smoking cessation and tobacco control, especially in the context of oncology meetings, should be an important focus.

The American Thoracic Society (ATS) has previously reported on the low proportion of tobacco control–related research presented at its annual meetings. In a review from 2002 to 2008, the total number of tobacco control presentations ranged from 103 to 148 accepted abstracts and educational sessions per year.^22^ However, in oncology scientific meetings, in which there are also strong associations between tobacco and cancer, the amount of content on tobacco control and smoking cessation is less well known. The educational content of scientific meetings is generally developed by the program educational committees, whereas abstracts are submitted by researchers and potential attendees. Gaining insight into the amount of educational and abstract content in oncology meetings may help to inform strategies to encourage tobacco control research and methods for the dissemination of tobacco control and smoking cessation–related (TCSCR) content at oncology meetings.

Here, we conducted a systematic scoping review to assess the amount of TCSCR research presented at recent major oncology meetings. Our aims were the following: (1) to determine the quantity of TCSCR content presented across major oncology meetings; and (2) to evaluate the characteristics of the abstracts and educational sessions presented.

## Materials and Methods

### Selection of Study Meetings

We conducted a scoping review using the methodological framework proposed by Arksey et al.^23^ Abstracts and educational sessions presented at 12 major international oncology meetings between January 2018 to July 2023 were reviewed. Major oncology meetings were selected for our analysis with the aim of exploring internationally representative meetings. The 12 meetings were on the basis of four unique themes to represent diverse topics and research focuses on oncology. As there are many major oncology meetings, the inclusion of meetings was decided on the basis of size, global representation, thematic relevance, and data availability. All were major international oncology meetings and included the following: (1) six discipline-focused meetings (American Society for Clinical Oncology [ASCO], European Society for Medical Oncology [ESMO], American Society for Radiation Oncology [ASTRO], European Society for Radiation Oncology [ESTRO], Society for Surgical Oncology [SSO], and European Society for Surgical Oncology [ESSO]); (2) two age group–specific meetings (the Society for Pediatric Oncology [SIOP] and Society for Geriatric Oncology [SIOG]); (3) two additional multidisciplinary meetings (the American Association for Cancer Research [AACR] and World Cancer Congress [WCC]); and (4) two disease site–specific meetings (the World Conference for Lung Cancer [WCLC] and the Multidisciplinary Head and Neck Cancers Symposium [MHNCS]). We specifically chose these two disease–site meetings because of the disease site’s strong association with tobacco, although WCLC includes not only lung cancer but also additional thoracic cancers (including thymic cancers and mesothelioma). Scientific abstracts were classified as content submitted by the potential attendees and educational sessions whose content was set by the conference educational committee.

### Search Query and Study Selection

We accessed conference proceedings, abstract books, online programs, and schedules for each meeting to obtain information on both the abstracts and educational sessions at each of the oncology meetings, including presentation titles, authors, and published abstracts. We used 10 keywords and MeSH terms to help identify TCSCR content, namely: “Tobacco,” “Cigarette,” “Nicotine,” “Pack Years,” “Pack-Years,” “Smoke,” “Quitting,” “Cessation,” “Bupropion,” and “Varenicline.”

Abstract and educational session eligibility were independently reviewed by two of nine reviewers (SC, MC, PC, AA, JD, WB, DC, RH, MK). Disagreements on eligibility were resolved by a third reviewer (LE). Eligible content needed to be relevant to tobacco control or smoking cessation and cancer. Eligible content had to be online and accessible, and written in English. Abstracts in which tobacco use was solely evaluated as a covariate or in which the primary focus was not on tobacco control or smoking cessation were excluded.

### Data Extraction

Data extracted from each abstract included the type of presentation (oral, poster, publication only), research focus (cancer risk, cancer outcomes, smoking cessation), disease site focus (including those that involved multiple disease sites or were disease site agnostic), research methods (clinicoepidemiologic, quality improvement or health services, basic science or translational, review studies), and the country of origin of the first author. The country of origin was further classified into high-income countries (HIC) or low-middle-income countries (LMIC) on the basis of the World Bank Country Classification.^24^

### Statistical Analysis

Descriptive statistics were used to characterize the frequency of both educational and abstract content and to summarize content characteristics. As meetings vary in size and scope and have different total numbers of educational sessions and accepted abstracts, the mean percentage of TCSCR content was calculated with all conference years weighted equally between meetings. Pearson’s chi-square or Fisher’s exact tests were performed to determine differences in the proportion of abstract characteristics between HIC and LMIC. TCSCR abstracts and educational sessions presented pre–coronavirus disease 2019 (COVID-19) (2018–2019) and post–COVID-19 (2022–2023) years were compared. We specifically excluded 2020 to 2021 as those years were when the pandemic was at its peak and had a significant impact on research and conference attendance. We also applied linear regression modeling to evaluate trends in the overall percentage of TCSCR abstract and education sessions over time. Pearson’s chi-square test was performed to compare the total proportions from pre- and post–COVID-19. *P* values less than 0.05 were considered statistically significant. All statistical analyses were conducted on IBM–Statistical Package for the Social Sciences version 29 (IBM SPSS Statistics, IBM Corp., Armonk, New York).

## Results

### Identification of Abstract and Educational Sessions

From a total of 145,830 educational sessions and abstracts, 5178 abstracts and educational sessions were initially identified using 10 key search terms and MeSH terms (Supplementary Table 1). There were 4430 abstracts and 208 educational sessions that were excluded after screening by reviewers on the basis of our inclusion and exclusion criteria. A total of 421 abstracts and 119 educational sessions presented at the 12 major oncology meetings between January 2018 to July 2023 met our final eligibility criteria.

### Tobacco Control and Smoking Cessation Content in Abstract Submissions

Table 1 presents the overall results for TCSCR content presented as abstracts at the 12 major oncology meetings. Overall, on average, TCSCR abstracts represented 0.52 (± 1.18%) of all accepted abstracts. There was some variation in the percentage of TCSCR abstracts between years, with the highest percentage being in 2018 (0.59%) and the lowest in 2021 (0.19%). Both WCC and WCLC had the largest mean percentages of TCSCR abstracts relative to all other meetings (WCC mean per meeting: 4.96 ± 3.77%; WCLC mean per meeting: 1.81 ± 0.77%). The meetings that were disease site–specific had the highest mean percentage of TCSCR abstracts (MHNCS mean per meeting: 1.29 ± 1.05%, WCLC mean per meeting: 1.81 ± 0.77%; overall mean per meeting: 1.64 ± 0.82% relative to all other meeting types). The pediatric and geriatric-focused meetings had the lowest mean percentage (SIOP mean per meeting: 0.00 ± 0.00%; SIOG mean per meeting: 0.14 ± 0.21%; overall mean per meeting: 0.05 ± 0.15%). The mean percentage of TCSCR abstracts presented at the additional multidisciplinary meetings was 1.54 (± 2.67%) (AACR mean per meeting: 0.32 ± 0.06%; WCC mean per meeting: 4.96 ± 3.77%).

**Table 1:** Total Numbers and Percentages of Included Tobacco Control and Smoking Cessation–Related Abstracts Across Oncology Meetings

Meetings	2018		2019		2020		2021		2022		2023		Mean ± SD
	n	%	n	%	n	%	n	%	n	%	n	%	
Discipline-focused meetings – Medical Oncology													
American Society of Clinical Oncology (ASCO)	8 5419	0.15%	8 5419	0.15%	13 5032	0.26%	4 5287	0.08%	9 5573	0.16%	11 6150	0.18%	0.17 ± 0.06%
European Society for Medical Oncology (ESMO)	3 2054	0.15%	6 2218	0.27%	3 2334	0.13%	6 2117	0.28%	3 2038	0.15%	a	a	0.20 ± 0.08%
Discipline-focused meetings – Radiation Oncology													
American Society for Radiation Oncology (ASTRO)	4 2253	0.18%	6 2444	0.25%	0 2562	0.00%	2 1645	0.12%	2 1707	0.12%	a	a	0.13 ± 0.09%
European Society for Radiotherapy & Oncology (ESTRO)	2 1586	0.13%	4 1902	0.21%	2 1700	0.12%	1 1699	0.06%	1 1578	0.06%	0 2359	0.00%	0.09 ± 0.07%
Discipline-focused meetings – Surgical Oncology													
Society of Surgical Oncology (SSO)	0 554	0.00%	0 577	0.00%	2 577	0.35%	0 560	0.00%	1 376	0.27%	3 571	0.53%	0.19 ± 0.22%
European Society of Surgical Oncology (ESSO)	0 461	0.00%	0 490	0.00%	1 165	0.61%	0 435	0.00%	0 529	0.00%	a	a	0.05 ± 0.27%
Age group–focused meetings													
International Society of Pediatric Oncology (SIOP)	0 840	0.00%	0 1411	0.00%	0 1099	0.00%	0 1171	0.00%	0 1390	0.00%	a	a	0.00 ± 0.00%
International Society of Geriatric Oncology (SIOG)	0 148	0.00%	1 215	0.47%	0 11	0.00%	0 159	0.00%	0 183	0.00%	a	a	0.14 ± 0.21%
Disease site–focused meetings													
World Conference on Lung Cancer (WCLC)	33 1660	1.99%	37 1232	3.00%	15 1250	1.20%	10 807	1.24%	17 1254	1.36%	a	a	1.81 ± 0.77%
Multidisciplinary Head and Neck Cancers Symposium (MHNCS)	7 314	2.23%	b	b	1 396	0.25%	b	b	4 217	1.84%	b	b	1.29 ± 1.05%
Additional multidisciplinary meetings													
American Association for Cancer Research (AACR)	25 7139	0.35%	22 5314	0.41%	13 4693	0.28%	12 4113	0.29%	15 6357	0.24%	26 7266	0.36%	0.32 ± 0.06%
World Cancer Congress (WCC)	55 700	7.86%	b	b	b	b	b	b	21 833	2.52%	b	b	4.96 ± 3.77%
Year Total	137 23,128	0.59%	86 21,016	0.41%	50 19,819	0.25%	35 17,993	0.19%	73 22,035	0.33%	40 16,346	0.24%	

*Note:* The numerators of n values represent the total number of tobacco control and smoking cessation–related abstracts. The denominators of the n values represent the total number of abstracts presented for the meeting. The percentages of tobacco control and smoking cessation–related content are out of all accessible abstracts presented for the meeting and meeting year.

a The meeting did not occur within our analysis time frame.

b The meeting did not occur.

The mean percentage of TCSCR abstracts presented at discipline-focused meetings overall was low at 0.15 (± 0.15%). Specifically, among medical oncology meetings, the average was 0.18 (± 0.07%) per meeting (ASCO mean per meeting: 0.17% ± 0.06%; ESMO mean per meeting: 0.20 ± 0.08%). Among radiation oncology meetings, the average was 0.11 (± 0.08%) per meeting (ASTRO mean per meeting: 0.13 ± 0.09%; ESTRO mean per meeting: 0.09 ± 0.07%) and among surgical oncology meetings, the mean amount of TCSCR content per meeting was 0.16 (± 0.24%) (SSO mean per meeting: 0.19 ± 0.22%; ESSO mean per meeting: 0.05 ± 0.27%).

The identified abstracts represented a range in content across smoking cessation research topics being presented at oncology meetings. These included basic science and translational research abstracts such as one exploring the role of *TBX2* subfamily in the development of lung adenocarcinoma using RNA sequencing and mouse models exposed to tobacco carcinogens,^25^ to those focused on health services research exploring the impact of implementing incentives through smoke-free zone certifications to support the smoke-free policies in Jordan.^26^ Additional abstracts focused on clinical and epidemiology aspects including a retrospective review evaluating the efficacy of chemoimmunotherapy on the basis of smoking status and programmed death-ligand 1 expression among patients with advanced NSCLC,^27^ and reviews including assessing the frequency of tobacco use reporting in cancer cooperative group clinical trials.^28^ These sample abstracts in our research type analysis show the heterogeneity in the scope of smoking cessation research and initiatives submitted across the oncology meetings.

### Tobacco Control and Smoking Cessation Content Among Conference Educational Sessions

Table 2 presents the results for TCSCR content presented as educational sessions at the major oncology meetings. For all meetings across all years, the overall mean percentage of TCSCR educational sessions per meeting was 0.54 (± 1.24%). The year 2019 had the highest overall percentage per meeting (0.85%) of TCSCR educational content presented across the 12 meetings, and 2023 had the lowest percentage (0.28%). The WCC and WCLC had the greatest mean percentage of TCSCR educational sessions per meeting relative to all other meetings (WCC mean: 3.48 ± 1.30%; WCLC mean: 3.15 ± 1.06%).

**Table 2:** Total Numbers and Percentages of Included Tobacco Control and Smoking Cessation–Related Educational Sessions Across Oncology Meetings

Meetings	2018		2019		2020		2021		2022		2023		Mean ± STDEV
	n	%	n	%	n	%	n	%	n	%	n	%	
Discipline-focused meetings – Medical Oncology													
American Society of Clinical Oncology (ASCO)	1 622	0.16%	0 769	0.00%	1 289	0.35%	0 442	0.00%	1 610	0.16%	2 554	0.36%	0.15 ± 0.16%
European Society for Medical Oncology (ESMO)	1 707	0.14%	0 716	0.00%	5 615	0.81%	1 672	0.15%	0 829	0.00%	a	a	0.20 ± 0.34%
Discpline-focused meetings – Radiation Oncology													
American Society for Radiation Oncology (ASTRO)	0 111	0.00%	0 126	0.00%	0 132	0.00%	0 122	0.00%	0 109	0.00%	a	a	0.00 ± 0.00%
European Society for Radiotherapy & Oncology (ESTRO)	0 307	0.00%	0 311	0.00%	0 244	0.00%	0 275	0.00%	0 297	0.00%	0 273	0.00%	0.00 ± 0.00%
Discipline-focused meetings – Surgical Oncology													
Society of Surgical Oncology (SSO)	0 84	0.00%	b	b	b	b	b	b	b	b	0 86	0.00%	0.00 ± 0.00%
European Society of Surgical Oncology (ESSO)	0 149	0.00%	0 160	0.00%	0 175	0.00%	0 166	0.00%	0 222	0.00%	a	a	0.00 ± 0.00%
Age Group–Focused Meetings													
International Society of Pediatric Oncology (SIOP)	b	b	b	b	0 461	0.00%	0 477	0.00%	0 603	0.00%	a	a	0.00 ± 0.00%
International Society of Geriatric Oncology (SIOG)	0 106	0.00%	0 46	0.00%	0 11	0.00%	0 105	0.00%	b	b	a	a	0.00 ± 0.00%
Disease site–focused meetings													
World Conference on Lung Cancer (WCLC)	19 521	3.65%	23 569	4.04%	8 425	1.88%	13 313	4.15%	13 582	2.23%	a	a	3.15 ± 1.06%
Multidisciplinary Head and Neck Cancers Symposium (MHNCS)	0 47	0.00%	c	c	0 62	0.00%	c	c	0 74	0.00%	c	c	0.00 ± 0.00%
Additional multidisciplinary meetings													
American Association for Cancer Research (AACR)	1 1338	0.07%	b	b	1 918	0.11%	6 1221	0.49%	3 1055	0.28%	5 1572	0.32%	0.26 ± 0.17%
World Cancer Congress (WCC)	12 380	3.16%	c	c	c	c	c	c	4 80	5.00%	c	c	3.48 ± 1.30%
Year Total	34 4372	0.78%	23 2697	0.85%	15 3331	0.45%	20 3793	0.53%	21 4461	0.47	7 2485	0.28%	

*NOTE:* The numerators of n values represent the total number of tobacco control and smoking cessation–related educational sessions. The denominators of the n values represent the total number of educational sessions presented for the meeting. The percentages of tobacco control and smoking cessation–related content are out of all accessible educational sessions presented for the meeting and meeting year.

a The meeting did not occur within our analysis time frame.

b The meeting content was not accessible or retrievable.

c The meeting did not occur.

The disease site–focused meetings had the highest mean percentage of TCSCR educational sessions relative to the other meeting types at 1.99 (± 1.84%) per meeting (WCLC mean per meeting: 3.15 ± 1.06%; MHNCS mean per meeting: 0.00 ± 0.00%). The age-related meetings did not have any TCSCR content across the time period. The additional multidisciplinary meetings combined had a mean percentage of 1.35 ± 1.94% per meeting (AACR mean: 0.26 ± 0.17%; WCC mean: 3.48 ± 1.30%) of TCSCR educational sessions. The discipline-focused meetings had a combined mean percentage of 0.00 (± 0.17%). The medical oncology meetings had an overall mean per meeting of 0.19 (± 0.24%) (ASCO mean per meeting: 0.15 ± 0.16%; ESMO mean per meeting: 0.20 ± 0.34%). The radiation oncology and surgical oncology meetings did not have any TCSCR education sessions.

### Temporal Trends of TCSCR Abstracts and Educational Content

**Figure 1:**
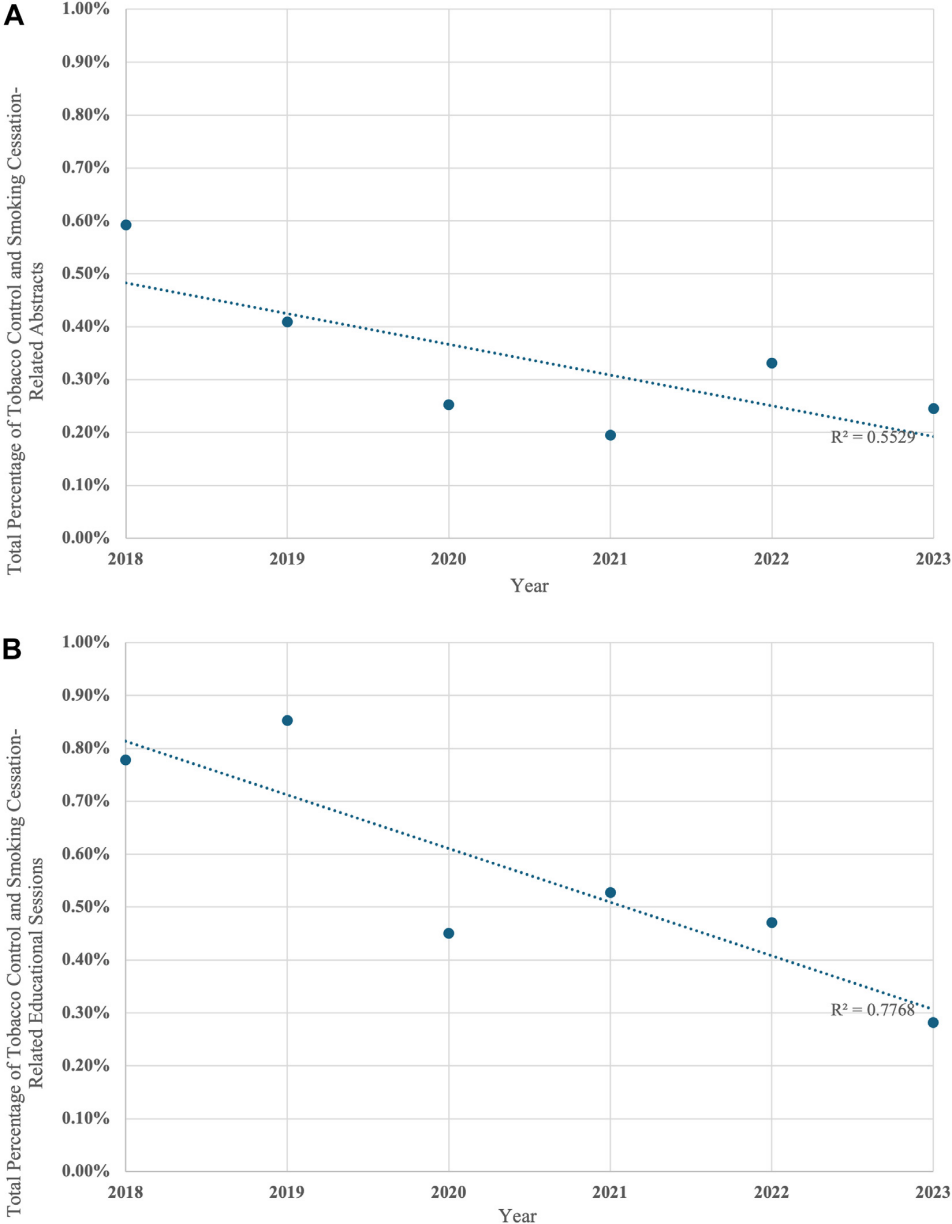
Total proportions of tobacco-related content presented by year. (*A*) Abstract presentations. (*B*) Educational sessions.

Figure 1 displays the temporal trend in TCSCR content from 2018 to 2023 across all meetings. Among abstracts, there was a nonsignificant trend toward a reduction in the overall percentage of TCSCR abstracts over the time period (r^2^ = 0.55, *p* = 0.09; Figure 1*A*). Similarly, among educational sessions, there was a significant reduction in TCSCR education sessions over the study period (r^2^ = 0.77, *p* = 0.02; Figure 1*B*). Comparisons of the total proportion of TCSCR content reveal a significant decrease from pre–COVID-19 to post–COVID-19 of abstracts (0.51% to 0.29%, *p* < 0.001) and educational sessions (0.81% to 0.40%, *p* = 0.002).

### Abstract Characteristics

**Figure 2:**
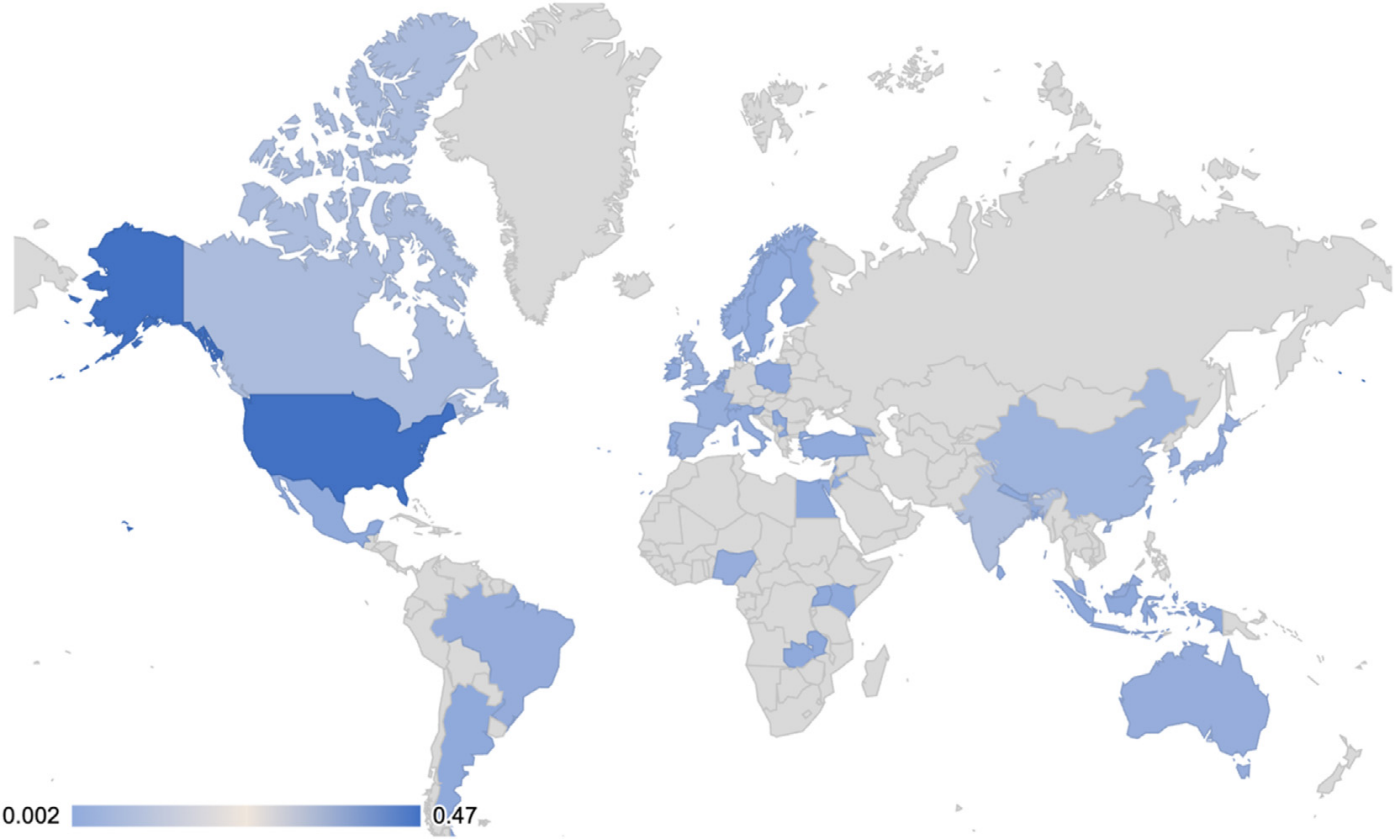
Heat map illustrating the geographic distribution of the countries represented by the first authors of tobacco-related abstracts. Darker blue represents higher concentrations of first authors originating from the respective country. Lighter blue represents lower concentrations.

Table 3 summarizes the distribution of the identified abstract characteristics. The majority (79%) of first authors were from HIC. Among abstracts, 40% reported on the impact of tobacco or smoking as a risk factor, 34% on smoking cessation, and 37% on the impact of tobacco on outcomes. Half (48%) of the abstracts were clinical-epidemiologic research studies; 29% were quality improvement or health services research studies; 21% were basic science and translational-focused; and 2% were systematic reviews. Two-thirds (65%) of the accepted TCSCR abstracts were presented as posters, 27% as oral abstracts, and 8% were publication-only abstracts. Over half (54%) of the first authors were based in North America, 21% from Asia, 18% from Europe, 3% Africa, 2% Australia, and 2% South America (Fig. 2).

**Table 3:** Summary of Included Abstract Characteristics

Variable	Subgroup	Total (n = 421)
Research Focus	Smoking as a cancer risk factor	39%
	Smoking cessation	34%
	Impact of smoking on cancer outcomes	26%
Session Type	Poster Abstract	65%
	Oral Abstract	27%
	Publication Only Abstract	8%
Research Type	Clinical and Epidemiologic	48%
	Quality Improvement and Health Services	29%
	Basic Science and Translational	21%
	Systematic Review	2%
Disease Site	Multiple and Nonspecific	38%
	Lung	38%
	Head and Neck	12%
	Gastrointestinal	4%
	Genitourinary	3%
	Breast	2%
	Hematologic	1%
	Skin	0.5%
	Central Nervous System	0.2%
	Gynecologic	0.2%
	Sarcoma	0.2%
Continent of First Authors	North America	54%
	Asia	21%
	Europe	18%
	Africa	3%
	Australia	2%
	South America	2%
Country Income Level of First Authors	High-Income Countries	79%
	Low-Middle-Income Countries	21%

Both the distribution of research focus (*p* = 0.004) and sessions (*p* < 0.001) were statistically significantly different among abstracts from HIC and LMIC (Table 4). HIC were more likely to have research focused on the impact of tobacco on outcomes, whereas LMIC focused more on smoking cessation. Also, HIC were more likely to have poster presentations, whereas LMIC were more likely to have oral presentations.

**Table 4:** Summary of Abstract Characteristics Between High-Income and Low-Middle-Income Countries

Variable	Subgroup	High-Income Country	Low-Middle-Income Country	*p* Value
Research Focus	Risk	40%	38%	0.004
	Outcomes	29%	15%	
	Smoking Cessation	31%	47%	
Session	Poster	72%	39%	< 0.001
	Oral	20%	54%	
	Published	8%	7%	
Research Type	Clinical and Epidemiologic	49%	46%	0.378
	Quality Improvement and Health Services	28%	33%	
	Basic Sciences and Translational	22%	17%	
	Review	2%	3%	

## Discussion

Smoking is a known risk factor for cancer, and continued tobacco use after a cancer diagnosis can worsen clinical outcomes. Despite the importance of smoking cessation in cancer care, little prominence is given to this topic at major oncology conferences. Through our scoping review, we identified that there were a limited number of tobacco control and smoking cessation–related abstracts and educational sessions across 12 major international oncology meetings. The WCC and WCLC had the highest mean percentage of education sessions and tobacco-focused abstracts, with most being submitted from HIC. To our knowledge, this is the first study formally measuring the quantity of TCSCR content presented at cancer conferences. These results suggest that oncology meetings may need to develop strategies to better highlight educational content related to tobacco control and smoking cessation and identify ways to encourage research submissions related to tobacco control and smoking cessation, especially from LMICs.

A previous review of tobacco-focused presentations and abstracts at the ATS conference from 2002 to 2008 revealed that the total number of abstracts and educational sessions per annual meeting ranged from 103 to 148 per year during the period of analysis.^22^ This is much greater than any of the meetings evaluated in our study. This is likely because of the strong association of tobacco use with other thoracic and respiratory diseases in addition to lung cancer, which is highlighted at ATS. In contrast, the total number of tobacco-related educational sessions presented at the ATS international conference was limited, ranging from 0 to 9 sessions per year over the time period, which was less than the number of educational sessions in some meetings in our analysis. However, this has not been formally measured among oncology meetings, where there are also strong associations with tobacco use and cancer risk and outcomes.

Despite the importance of tobacco control and smoking cessation in cancer care, the relative amount of content remains low compared with other topics and is also less than that at ATS meetings, in which the primary focus is on pulmonary health and disease. Similarly, our review found that most of the smoking-related abstracts across meetings focused on thoracic cancers and head and neck cancers, which are disease sites with strong associations with tobacco. However, smoking is relevant to many other cancers, including bladder, kidney, and pancreatic cancers, and also has important impacts on cancer treatments and outcomes across disease sites.^6^^,^^8^^,^^29^ Thus, it is important to encourage TCSCR research to expand its focus and content to other disease sites beyond the chest and head and neck sites. Our findings revealed a significant decrease in the total proportion of TCSCR content across all meetings, both over time and post–COVID-19, compared with the pre–COVID-19 meeting years. This suggests that the pandemic likely created challenges in conducting and disseminating smoking-related oncology research. During this time, many sessions within major meetings also shifted to focus on understanding the impact of COVID-19 and cancer.^30^ Moreover, submission and publication rates may have declined because of shifts in research funding to COVID-19–related topics and limitations in research capacity during lockdowns.^31^, ^32^, ^33^ As a result, strategies are needed to try to help improve both educational and research content related to tobacco control and smoking cessation in oncology meetings.

Various strategies can be piloted to improve TCSCR content in oncology meetings. In response to the low tobacco control-focused activity at the ATS conferences, organization-wide initiatives were undertaken to increase tobacco control programming, including the development of tobacco research awards.^22^ Other conferences have also adopted this approach, including the International Association for the Study of Lung Cancer.^34^ Similarly, the International Union Against Tuberculosis and Lung Disease selected tobacco control as a key theme for their 45^th^ Union World Conference on Lung Health. By making this the theme of the meeting, it attracted a record number of smoking-related abstracts and staged a large number of educational sessions.^35^ The implementation of topic-focused tracks and awards has been found to be effective in promoting more abstract submissions in an area selected for focus, thereby ensuring topic representation in conference programs and fostering research in the selected field. This has been seen with other topics such as diversity, equity, and inclusion,^36^, ^37^, ^38^ in which strategies such as having dedicated educational and abstract sessions, focused committees, and implementation of meeting awards and programs have helped to support an increase in diversity, equity, and inclusion content over time.^39^, ^40^, ^41^, ^42^

Translating some of the learnings from these other meetings to tobacco research and education may help to promote its growth across oncology meetings. However, tobacco control and smoking cessation tracks have not been implemented across many oncology scientific and education programs, despite tobacco control and smoking cessation playing an important role in both primary and secondary cancer prevention. This may be because of the advancements in other areas of oncology, including cancer treatments and clinical trials, and also the grouping of tobacco control and smoking cessation topics with other areas, including survivorship and cancer prevention.^43^ At times, smoking cessation content is also often incorporated into other tracks, including health services and quality improvement, which may also be smaller tracks at major oncology meetings with fewer sessions in conference programs.^44^ Creating tobacco research awards for trainees and professionals from developing nations may also help to further encourage the presentation of tobacco-related research at major oncology meetings. Meetings such as the WCLC, which focuses on thoracic cancers, have implemented tobacco control and smoking cessation awards for trainees from developing countries to lower the barrier to conference attendance and to encourage more global presentations. WCLC also has dedicated tobacco control tracks and sessions, which may explain the relatively greater content compared with other meetings.^45^

Given the importance of tobacco control and smoking cessation for patients with cancer and the current gaps in tobacco research, it is important to highlight more tobacco-related content at oncology meetings and further encourage research within the field. Many oncology organizations, including AACR and ASCO, recognize the need for more tobacco research to better understand smoking from a scientific and policy perspective.^46^, ^47^, ^48^ However, there is also a growing need for more research on newly emerging smoking-related habits, including cannabis and e-cigarette use and their relation to cancer and its treatments.^20^^,^^21^^,^^49^ With many new cancer treatments, especially involving immunotherapy and targeted therapies, the impact of tobacco on treatment-related outcomes, including toxicities, is less well known.^50^, ^51^, ^52^ Part of this is because of the lack of routine collection and analysis of tobacco use in cancer clinical trials.^53^, ^54^, ^55^, ^56^ To address this, the integration of dedicated tobacco control and smoking cessation tracks can promote further research and dissemination of these important findings, which may have broad implications across cancer care. Creating dedicated tracks can help ensure the representation of smoking-related content. However, dispersing some tobacco-related content into other tracks and sessions may also be beneficial, as this content can reach broader audiences who may not be attending tobacco-related sessions. This is a strategy that has been adopted by WCLC.

Our study has limitations. Given the use of publicly accessible conference abstracts, proceeding files, and program search tools, there were potential conference abstracts and educational sessions that may have been missing or not captured in the online resources. The total number of abstracts and educational sessions was not released by the meetings and required manual counting of the sessions that were accessible, which may not be fully accurate. Furthermore, the COVID-19 pandemic created challenges for conference organization and attendance. Whereas some of the conferences we analyzed had conferences running virtually during the COVID-19 pandemic, other conferences may not have occurred during this time. The absence or reduced capacity of a meeting may have impacted the overall percentage of abstracts and educational sessions, including those related to tobacco. Authors of abstracts may have also opted not to submit their abstracts during this period, and their research programs related to tobacco and cancer may have been impacted by the pandemic. In addition, we only included abstracts and educational sessions written and presented in English, which may have deterred presentations from non-English speaking regions. Furthermore, our scoping review only focused on 12 major oncology meetings, but there are many other cancer conferences. Thus, our results may be subject to selection bias because of the limited selection and analysis of oncology meetings, which may not be representative across all meetings. However, we selected large meetings with a broad perspective and many tracks and sessions, and specific disease site meetings directly related to tobacco. We suspect that other large oncology meetings may have similar percentages of TCSCR content in their meetings or potentially less. Our study explored the publication rates of conference abstracts, which did not account for the TCSCR abstracts that may have been submitted but were not accepted. Future analysis should investigate the TCSCR abstract acceptance rate at oncology conferences and the publication rates of the abstracts to full-text articles.

In summary, we found that TCSCR abstracts and educational sessions were limited at major oncology meetings. The WCC and WCLC had a relatively higher percentage of tobacco-related content. Most tobacco-focused abstracts originated from HIC. Considering the global importance of smoking cessation on cancer risk and cancer outcomes, conference organizing committees should consider implementing strategies to encourage a greater number of TCSCR abstract submissions, abstract sessions, and educational sessions to advance the global understanding of the impact of tobacco on the delivery of high-quality cancer care.

## CRediT Authorship Contribution Statement

**Sun Choi:** Conceptualization, Data curation, Formal analysis, Investigation, Methodology, Project administration, Resources, Supervision, Validation, Visualization, Roles/Writing -original draft, Writing - review & editing.

**Monisha Chawla:** Investigation, Writing - review & editing.

**Payton Catherwood:** Investigation, Writing - review & editing.

**David Chen:** Investigation, Writing - review & editing.

**Ryan S. Huang:** Investigation, Writing - review & editing.

**William Boateng:** Investigation, Writing - review & editing.

**Jennifer Do:** Investigation, Writing - review & editing.

**Maha Khan:** Investigation, Writing - review & editing.

**Abdulrahman Alghabban:** Investigation, Writing - review & editing.

**Naa Kwarley Quartey**: Project administration, Writing - review & editing.

**Srinivas Raman**: Supervision, Writing - review & editing.

**Meredith E. Giuliani:** Conceptualization, Investigation, Methodology, Supervision, Validation, Visualization, Roles/Writing - original draft, Writing - review & editing.

**William K. Evans:** Conceptualization, Investigation, Methodology, Project administration, Resources, Supervision, Validation, Visualization, Roles/Writing - original draft, and Writing - review & editing.

**Lawson Eng:** Conceptualization, Data curation, Formal analysis, Funding acquisition, Investigation, Methodology, Project administration, Resources, Supervision, Validation, Visualization, Roles/Writing - original draft, and Writing - review & editing.

## Disclosure

The authors declare no conflicts of interest.
