# Supplementary material for: Tobacco Control and Smoking Cessation–Related Content in Oncology Meetings: A Systematic Scoping Review
Source: JTO Clin Res Rep. 2025 Jul 3;6(11):100874. doi: 10.1016/j.jtocrr.2025.100874 (PMC12550121; doi:10.1016/j.jtocrr.2025.100874)
Supplement: Supplementary Figure [file mmc1.docx]

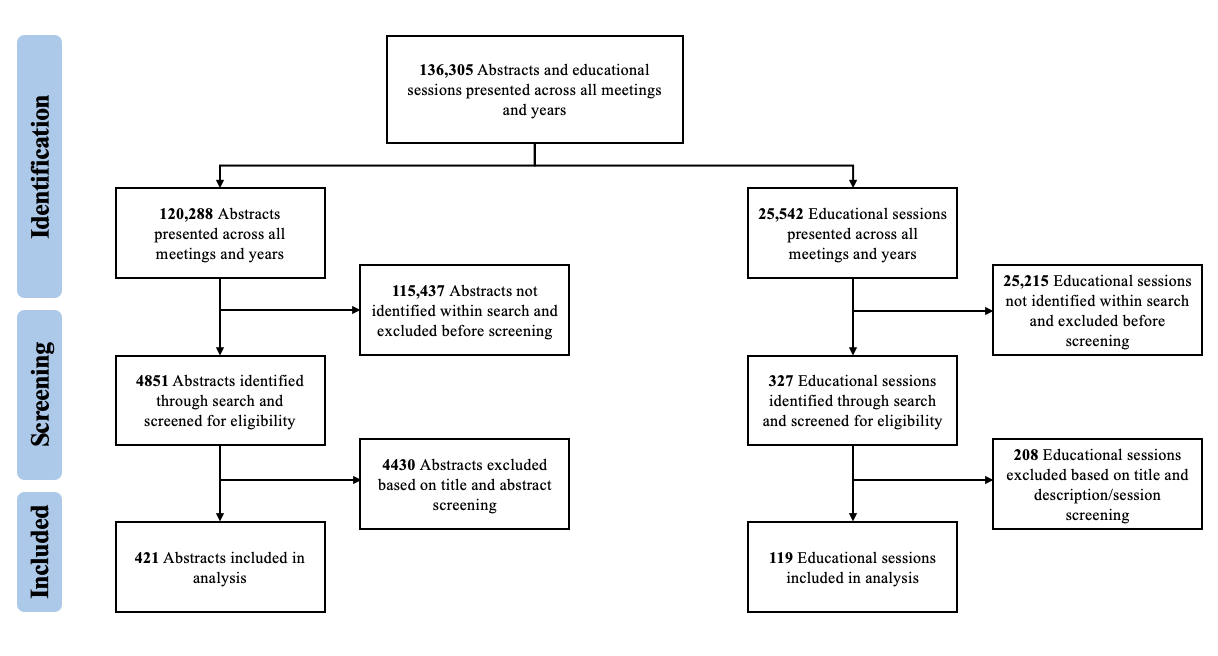


**Supplementary Figure 1.** Modified PRISMA flow diagram summarizing literature search and selection process.
